# A low cost, high fidelity nerve block model

**DOI:** 10.1186/s13089-014-0012-2

**Published:** 2014-08-13

**Authors:** Scott Sparks, David Evans, Don Byars

**Affiliations:** 1 Department of Emergency Medicine, Emergency Ultrasound Section, St. Luke’s University Health Network, 744 Ostrum Street, Bethlehem 18015, PA, USA; 2 Department of Emergency Medicine, Virginia Commonwealth University Health System, 1250 East Marshall Street, Richmond 23298, VA, USA; 3 Department of Emergency Medicine, Eastern Virginia Medical School, Raleigh Building, Suite 304, 600 Gresham Drive, Norfolk 23507, VA, USA

**Keywords:** Regional anesthesia, Ultrasound, Phantom model, Nerve block

## Abstract

**Background:**

We have constructed a simple, inexpensive simulation model for ultrasound guided nerve blocks. To date there are no low cost, high fidelity models for nerve block simulations. The models that do exist are expensive and vaguely resemble actual anatomy. As ultrasound guided nerve blocks become more common in medical education it is essential to develop better training models to help increase the comfort level of the individual provider and increase the chances for success during live-patient procedures [Anaesth Intensive Care 37: 824-829, 2009].

**Methods:**

The nerve block model was produced with a single pork loin with pressure-injected ultrasound gel through both CAT 5 cable and IV tubing inserted length-wise into the pork loin.

**Results:**

Our nerve block model had a realistic, life-like feel simulating human tissue.

**Conclusion:**

This ultrasound nerve block model was inexpensive with life-like feel allowing resident trainees to develop more confidence and tactile skill to increase the chance for success.

## Background

For decades regional anesthesia has traditionally been taught using anatomic guidance where the patient was the model for trainees [1],[2]. In the modern era of medicine this is no longer an acceptable practice [1],[3]. Previous studies have shown that after simulation training, the novice can be more comfortable and more successful in executing the neuraxial blocks. First reported by La Grange and colleagues in 1978 as a way to decrease the complication rate of supraclavicular brachial plexus blocks [4]. Since then innumerable studies have shown better avoidance of side effects, reduction in medication load, faster onset of anesthesia, longer duration of blocks, and improved quality of blocks [5]. Despite the known advantages of simulated nerve blocks, the models have primarily been expensive low fidelity gelatin-type models.

Given the importance of simulation of training and the known improved outcomes, we developed an easy to create ultrasound model that can facilitate regional nerve blocks, as well as intravenous access [2]. The procedure for creating the phantom model are in the Appendix.

## Methods

**Figure 1:**
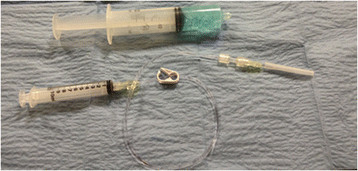
Supplies needed.

**Figure 2:**
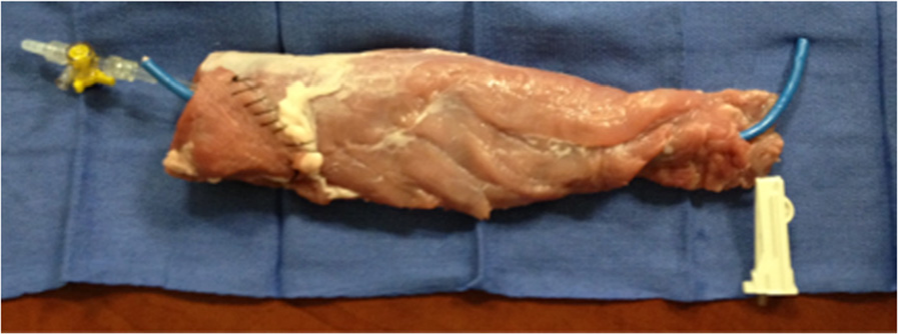
Completed nerve block model.

Using a similar method for epidural nerve block, we used a block of pork loin, CAT 5 ethernet cable, IV tubing, and ultrasound gel to create the phantom. Using a method for preservation previously described [3], we used hand sanitizer to preserve the pork loin overnight. Once preserved, we used long Kelly clamps to length-wise puncture the pork loin a single time through the center to grasp the ethernet cable and the intravenous tubing. Afterward, both the IV tubing and ethernet cable were pressure injected with ultrasound gel until it was expressed from the free end. We then clamped the ends of the IV tubing and ethernet cable with straight Kelly clamps to contain the gel Figures 1 and 2.

## Results and discussion

This produced a high fidelity model that was used multiple times for regional neuraxial blocks. The tissue was kept viable for a couple of weeks, as long as cooled afterward and kept wrapped to maintain the preservation effect. The total cost was nearly $25 USD. The low cost and reproducibility of the model make it an easy and cost-effective method of simulation.

**Figure 3:**
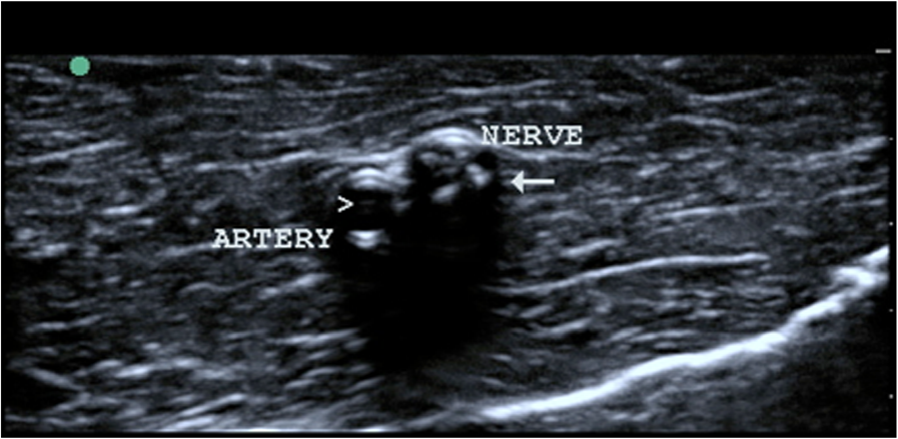
Ultrasonographic image of the nerve and artery.

**Figure 4:**
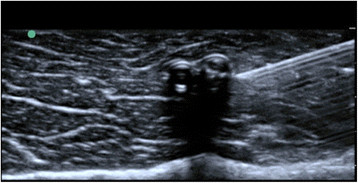
Ultrasound guided nerve block.

Although the model produced a great simulation model and prolonged use, it is limited by a definite shelf life when compared to commercial phantom models. However, this pork-loin model did give a more realistic tactile feel when compared to gelatine-type phantoms as well more realistic visual imaging during dynamic neuraxial block practice. We hope the use of high fidelity simulators for ultrasound guided regional anesthesia will lead to greater provider confidence and ultimately increased patient safety Figures 3 and 4.

## Conclusions

This nerve block model produced a life-like simulation for nerve block and vascular access. Using tools and equipment readily available, the pork loin was used repeatedly by resident trainees. The tissue felt life-like during the initial skin puncture and through the fascial planes. The CAT5 “nerve” was a good simulation for injecting local anesthetic and produced the “donut sign” of hypo echoic fluid around the nerve. Because the “nerve” was metallic wiring, it produced some posterior shadowing that limited the ability to visualize the posterior site of needle insertion or fluid spread; however, injecting the local anesthetic was visualized along the plane of the “nerve” similar to human tissue. Vascular access was practiced with hollow-bore metal needles to allow the feel of fluid aspiration and visualization and repositioning of the needle during dynamic ultrasound. Plastic vascular catheter placement was limited by the thickness of the IV tubing used.

Despite these limitations to posterior visualization and plastic catheter placement, this model will allow safe, more life-like, pain-free practice of neuraxial block and vascular access before attempting on patients to improve the chances for success.

## Appendix

Pork-Loin Neuraxial Model

Ingredients:

Store-bought Pork Loin

1 CAT 5 ethernet cable cut longer than Pork Loin

1 Peripheral IV tubing cut longer than Pork Loin

10 cc syringe and Starter IV connector to pressure inject

Ultrasound gel

1 Straight Kelly Clamp

1 Long Kelly Clamp

## Competing interests

The authors declare that they have no competing interests.

## Authors' contributions

All authors contributed to model design, execution, and manuscript preparation. All authors read and approved the final manuscript.
